# Membrane Separation for Rare Earth Elements (A Review)

**DOI:** 10.3390/membranes16020069

**Published:** 2026-02-19

**Authors:** Aaron T. Ben-Elijah, Tammy M. Lutz-Rechtin, S. Ranil Wickramasinghe, Xiaoyu Wang

**Affiliations:** Ralph E. Martin Department of Chemical Engineering, University of Arkansas, Fayetteville, AR 72701, USA

**Keywords:** membrane, separation, rare earth elements, REEs

## Abstract

Rare earth elements (REEs) are increasingly critical for advanced technologies like high-tech electronic devices, electric vehicles, catalysts, and supercapacitors. However, separating and purifying the REEs is challenging due to their similar physicochemical properties, such as ionic sizes and oxidation states. Traditional methods like solvent extraction require extensive use of organic solvents, involving multiple stages that generate large volumes of acidic liquid wastes. This article introduces membrane separation technologies as a more efficient approach that minimizes waste generation and offers higher selectivity and recovery rates in a single step. Membrane separation methods utilize free energy gradients and differences in ionic size or material affinity to selectively reject or allow ion adsorption and diffusion through the membrane pores. In this review paper, we critically evaluate recent advancements in the development and implementation of membrane-based systems and focus on exploring different membrane materials for REE separation, including polymer inclusion membranes, ion-imprinted membranes, nanofiltration membranes, electrodialysis membranes, metal-organic frameworks, and supported liquid membranes. The advantages, potential challenges, and technical issues with implementing these technologies are discussed, and possible areas for improvement and insights for further research are presented.

## 1. Introduction

**Figure 1:**
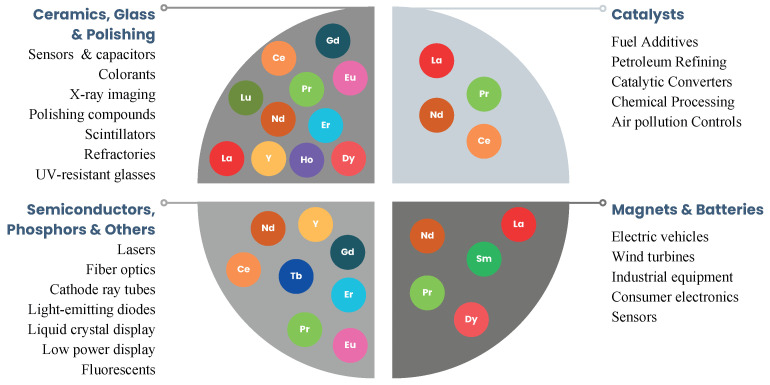
Summary of REEs applications across industries [3,5,8,9,10].

Rare earth elements (REEs), comprising the lanthanides, yttrium, and scandium, have become increasingly important due to their wide application in advanced technologies. Their uses include electronic devices, electric motors, catalytic converters, medical diagnostic equipment, and military guidance systems, as summarized in Figure 1 [1,2,3,4]. Their increasing economic importance, along with supply chains concentrated in a limited number of countries, has led to their classification as critical materials [5,6,7].

**Figure 2:**
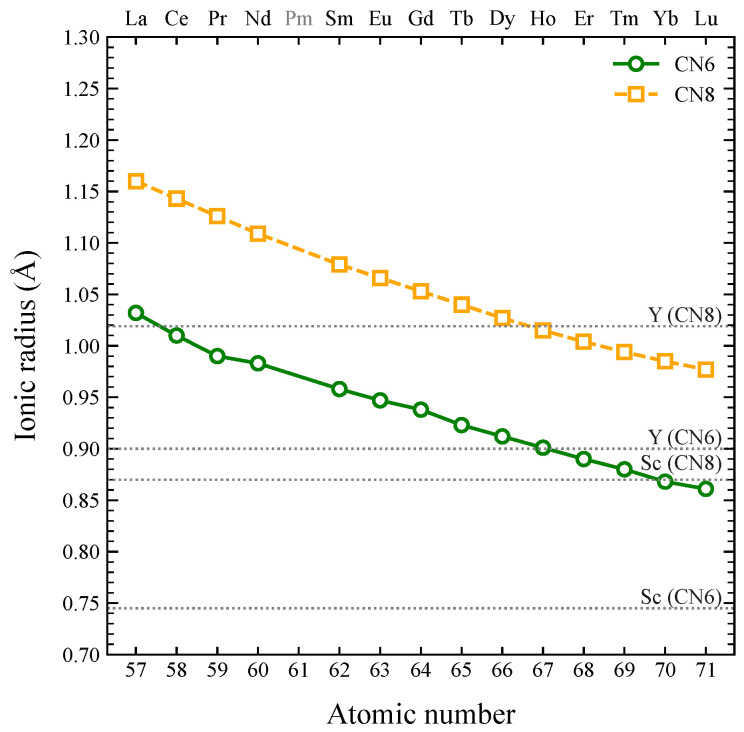
Plot of ionic radius versus atomic number for the trivalent lanthanide elements (La-Lu), Y and Sc, for coordination numbers (CN) 6 and 8 [13].

The importance of REEs in modern technologies arises from their unique electronic configurations, which results in remarkable magnetic, optical, and chemical properties [4,11]. Each REE has two outer s-orbital electrons, with the 3d, 4d, 4f, and 5d orbitals progressively filled (see Table 1), producing the *lanthanide contraction* from La to Lu (see Figure 2) [12]; Y and Sc are often grouped with the lanthanides due to similar ionic and chemical behavior [13,14]. In trivalent ions, shielded 4f electrons allow f-f electronic transitions responsible for optical and luminescent behavior [15,16], while unpaired 4f electrons result in paramagnetism and magnetocrystalline anisotropy [17]. Chemically, REEs act as strong reducing agents and exhibit high ionic potentials, accounting for their Lewis acidity and catalytic activity [18,19,20]. These electronic and reactive characteristics collectively underpin the critical roles of REEs in modern technologies.

Despite their importance in a variety of applications, the separation of REEs remains challenging due to their physicochemical similarity [21]. While this similarity enables the collective extraction of REEs as a group, it makes the separation and isolation of individual elements particularly challenging [22].

**Table 1:** Chemical properties of the rare earth elements.

Element	Symbol	Atomic Number	Possible Oxidation States [23]	Ground State Configuration [13]
Lanthanum	La	57	+3	[Xe]5d^1^6s^2^
Cerium	Ce	58	+3, 4	[Xe]4f^1^5d^1^6s2
Praseodymium	Pr	59	+3, 4	[Xe]4f^3^5s^2^
Neodymium	Nd	60	+3	[Xe]4f^4^6s^2^
Promethium	Pm	61	+3	[Xe]4f^5^6s^2^
Samarium	Sm	62	+2, 3	[Xe]4f^6^6s^2^
Europium	Eu	63	+2, 3	[Xe]4f^7^6s^2^
Gadolinium	Gd	64	+3	[Xe]4f^7^5d^1^6s^2^
Terbium	Tb	65	+3, 4	[Xe]4f^9^6s^2^
Dysprosium	Dy	66	+3	[Xe]4f^10^6s^2^
Holmium	Ho	67	+3	[Xe]4f^11^6s^2^
Erbium	Er	68	+3	[Xe]4f^12^6s^2^
Thulium	Tm	69	+2, 3	[Xe]4f^13^6s^2^
Ytterbium	Yb	70	+2, 3	[Xe]4f^14^6s^2^
Lutetium	Lu	71	+3	[Xe]4f^14^5d^1^6s2
Scandium	Sc	21	+3	[Ar]3d^1^4s^2^
Yttrium	Y	39	+3	[Kr]4d^1^5s^2^

## 2. Current Methods for REE Separation

The various high-tech applications of REEs often require high levels of purity [24]. However, in nature, they are typically found together within minerals, such as bastnaesite, monazite, ion-adsorption minerals, and yttrium phosphorite [25]. The process of converting these minerals into high-value materials mainly involves four stages: (1) mining and concentrating ores, (2) processing and separation of the ores to obtain groups of REEs or an individual REE, (3) production of metals and alloys, and (4) integration of the materials into different high-tech applications [19].

The traditional methods for separating concentrated REE ores include fractional crystallization, precipitation, and selective oxidation/reduction [8,26]. Fractional crystallization exploits the differing solubility of REE salts in solvents like dimethyl ether. By adjusting specific conditions such as temperature or pH, the least soluble species crystallize out of solution [27,28,29]. This method is particularly effective for separating lanthanides at the lower end of the series. A key example is the separation of the double nitrate $La{\left(NO_{3}\right)}_{3}\cdot 2NH_{4}NO_{3}\cdot 4H_{2}O$ from Pr and other trivalent lanthanides after removal of $Ce^{4+}$ [26].

Precipitation involves altering the solution’s pH or adding reagents to form insoluble compounds with specific anions, such as carbonates or phosphates [30]. This process exploits differences associated with ionic radii and typically follows systematic solubility trends across the REE series, correlated with decreasing ionic radii [26]. Selective oxidation/reduction separates REEs based on changes in oxidation state. For example, Ce_3+_ can be oxidized to Ce_4+_, which precipitates as hydrated cerium(IV) oxide, whereas Eu_3+_ can be reduced to Eu_2+_, allowing it to be removed as sparingly soluble Eu(II) sulfate. This approach effectively isolates specific REEs from other trivalent rare earths [31,32]. While effective, these methods are labor-intensive. Owing to low single-step separation factors, achieving high-purity products may require repeated fractional precipitation or equilibrium stages, with reported production cycles ranging from tens to thousands [31]. Consequently, such approaches are often less favored than newer, streamlined techniques such as solvent extraction.

**Figure 3:**
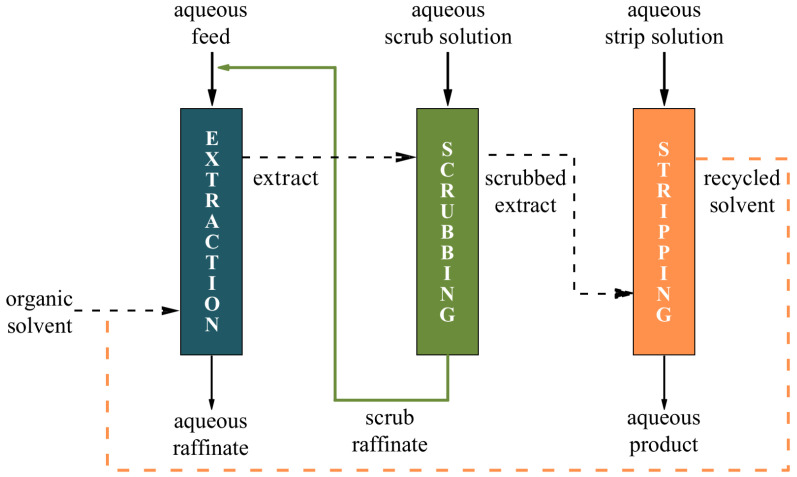
Solvent extraction flowchart.

Solvent extraction (SX) is the predominant industrial method for REE separation due to its tunable selectivity and scalability [33]. SX separates compounds based on their different solubilities in two immiscible liquids, mainly an aqueous phase and an organic solvent [34]. Figure 3 illustrates a typical SX process. The SX process involves three stages: extraction, scrubbing, and stripping. During extraction, REE ions are transferred from the aqueous phase to the organic solvent using an extractant; scrubbing subsequently removes co-extracted impurities; and stripping recovers the REEs back into an aqueous phase, allowing solvent reuse [35].

A key limitation of traditional SX methods is their low selectivity when separating adjacent REEs [36]. Due to similar chemical properties of REEs, their distribution coefficients and separation factors differ slightly [37,38]. As a result, achieving high purity requires multiple extraction and scrubbing stages. These processes generate large volumes of acidic aqueous waste and organic solvents, require high consumptions of expensive reagents and extractants, and raise significant sustainability concerns [39,40,41]. Furthermore, at an industrial scale, the low flow rate of the organic phase leads to poor dispersion in the aqueous phase. This limitation reduces interfacial contact and the specific surface area available for extractant-metal interactions, thereby increasing mass-transfer resistance and prolonging residence times. As a result, additional extraction stages are required to overcome these limitations [42].

The limitations of these conventional methods underscore the need for alternatives that require fewer separation stages, use less organic solvent, generate minimal acidic waste, and maintain high selectivity. Membrane-based separation methods have thus emerged as a promising solution. The following section outlines these techniques and their potential to address these limitations.

## 3. Membrane-Based Methods for REE Separation

Membrane separation methods have gained increasing attention as greener and more efficient alternatives for the REE separation [43]. They are preferred over traditional separation techniques, like solvent extraction, because they offer higher selectivity, good tunability, lower chemical consumption, minimal waste generation, and improved compatibility with continuous operations [44,45]. Therefore, they can reduce environmental footprints, and offer high yield and purity levels [46].

Membrane-based separations are typically achieved through three main mechanisms: (1) *carrier-mediated transport* via reversible complexation with mobile or fixed carriers embedded in the membrane matrix; (2) *selective adsorption* of ions onto fixed active or recognition sites (e.g., imprinted cavities) primarily on the membrane surface or within pores; and (3) *size- or charge-based rejection* across semipermeable membranes without specific chemical binding [45]. Although conceptually distinct, these mechanisms can overlap, as in carrier-mediated transport, which involves an initial adsorption/complexation step. Similarly, certain membrane architectures, such as metal-organic framework (MOF)-based membranes, can employ one or more of these mechanisms, depending on factors like pore structure, surface functionalization, and specific application [47].

### 3.1. Supported Liquid Membranes

Supported liquid membranes (SLMs) are liquid membrane systems in which an organic solvent is immobilized within the pores of a microporous solid support by capillary forces [48,49]. The immobilized liquid acts as a separation media between two aqueous phases (feed and stripping), establishing a concentration, pH, or electrochemical potential gradient that drives solute transfer. The organic solvent usually contains a ligand that selectively binds a target REE from the feed solution at the feed-membrane interface [50]. The resulting REE-ligand complex diffuses through the liquid-filled pores to the membrane-stripping interface, where a reverse reaction regenerates the ligand and releases the metal ions into the stripping solution. The regenerated ligand then diffuses back through the membrane, repeating the cycle [51]. This simultaneous transport of REE via complex formation and counter-migration of cations is referred to as *coupled facilitated counter-transport*, a phenomenon that enhances both the efficiency and selectivity of SLMs [50,52].

The transport of REE ion in SLMs resembles that of conventional SX processes, but with key differences [53]. SX is an equilibrium process governed by interfacial reaction kinetics and mass-transfer limitations due to phase mixing [54]. In contrast, REE transport in SLMs is a dynamic nonequilibrium process (uphill transport), primarily controlled by diffusion and REE partitioning at the feed-membrane and membrane-strip interfaces [48]. Moreover, SLMs integrate the extraction and stripping stages of SX into a single unit, providing a higher interfacial surface area and reducing the volume of organic solvent required for separation [55].

**Figure 4:**
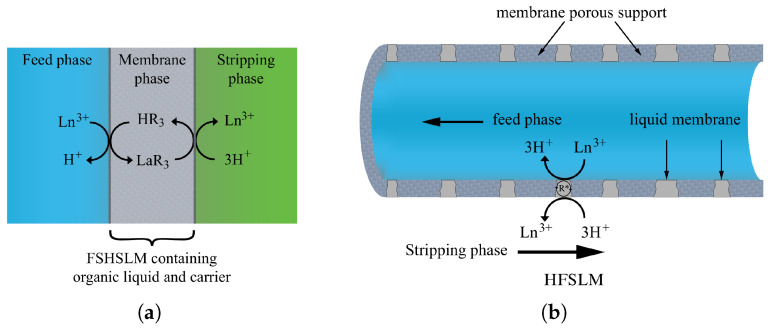
Schematic representation of a typical supported liquid membrane: (**a**) Flat-sheet supported liquid membrane (FSHSLM) (**b**) Hollow fibre supported liquid membrane (HFSLM). Where $La^{3+}$ denotes REE ion; $HR_{3}$ and R* represent extractants such as Cyanex 272; $LaR_{3}$ refers to metal-extractant complex [57,58].

Based on the configuration of the membrane support, SLMs can be further classified into flat-sheet supported liquid membranes (FSHSLMs) and hollow-fiber supported liquid membranes (HFSLMs) (Figure 4). FSHSLMs typically utilize polytetrafluoroethylene (PTFE) or polyvinylidene fluoride (PVDF) supports, while HFSLMs use polypropylene fibers. Particularly, HFSLMs offer larger surface areas, shorter diffusion paths, higher mass transfer coefficients, and lower solvent loss due to the capillary retention of the organic phase [51,56]. As a result, HFSLMs generally exhibit better selectivity and stability compared to flat-sheet systems.

Compared to multi-step SX processes, SLMs provide better extraction efficiencies in a single step. Some representative studies focusing on SLMs have been summarized in Table 2. Ni’am et al. [59] reported that HFSLMs using di(2-ethylhexyl)phosphoric acid (D2EHPA) in isopar-L extracted 58.6% of Nd, 98.5% of Dy, and 85.6% of Pr from waste permanent magnets, with corresponding recovery rates of 63.1%, 15.2%, and 56.3%. The extraction efficiencies and selectivity of SLMs are strongly influenced by the carrier type and operating conditions. For example, Wannachod et al. [60] achieved 98% extraction and 95% stripping of Nd^3+^ using HFSLMs at pH 4.5 with 0.5 M 2-ethylhexyl phosphonic acid mono-2-ethylhexyl ester. In another study, Wannachod et al. [61] reported up to 98% extraction and 95% stripping of Nd^3+^ from monazite leach using 0.5 M Di-nonyl phenyl phosphoric acid (DNPPA) and 3 M $H_{2}SO_{4}$. Synergistic ligands, such as Cyanex 272 and TBP, further improved selectivity for Y^3+^ from REE mixtures, achieving 70% extraction and 55% stripping [58].

Supported liquid membranes (SLMs) offer several notable advantages, as highlighted by Argurio et al. [50]. They provide a constant and well-defined effective contact area that is independent of fluid flow rates. Therefore, only small volumes of organic solvent are required, as the organic phase is immobilized within the membrane pores, and the extractant is used cyclically, further reducing reagent consumption. Moreover, the non-dispersive contact between the feed, membrane, and strip phases simplifies operation by eliminating additional separation steps and preventing emulsion formation. Continuous REEs release into the strip phase also maintains a strong driving force and minimizes limitations imposed by equilibrium constant.

Despite these advantages, SLMs still face significant challenges that hinder their industrial-scale application. For example, solvent loss caused by emulsion formation or pressure differences can result in low membrane stability. This instability reduces solute flux and selectivity over time, requiring solvent replenishment, membrane cleaning, or membrane replacement [63,64]. Membrane-based mass transfer resistance can also diminish separation performance. However, this can be mitigated by optimizing solute concentration gradients, employing hydrophobic supports with small pore size, or stabilizing membranes using interfacial polymerization layers [53,65,66].

### 3.2. Polymer Inclusion Membranes

**Figure 5:**
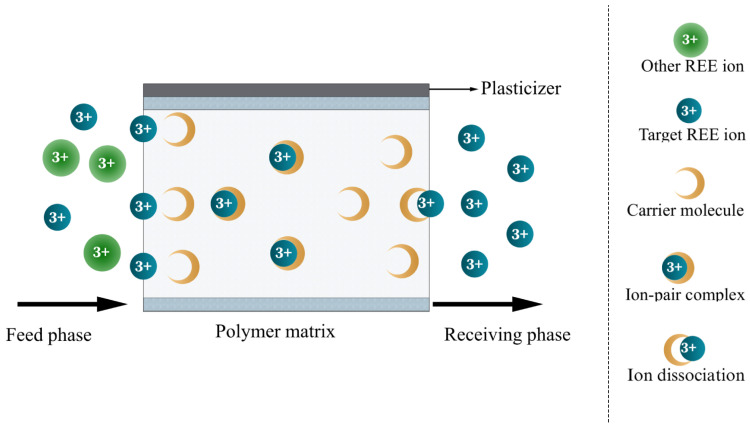
Schematic representation of a typical polymer inclusion membrane.

Polymer inclusion membranes (PIMs) are composite liquid membranes in which an ion-selective carrier is immobilized within a solid polymer matrix [67]. A typical PIM consists of three main components: a polymer matrix, an extractant, and an optional plasticizer (see Figure 5). The *solid* polymer matrix immobilizes the extractant and strengthens the mechanical property of the membrane [68]. Common polymer materials include cellulose triacetate (CTA), polyvinyl chloride (PVC), polytetrafluoroethylene (PTFE), and polyvinylidene fluoride (PVDF) [69,70]. Transport in PIMs primarily occurs through coordination between the extractant and REE ion [71]. Therefore, the extractant plays a central role in determining separation performance by enabling the selective transport of REEs across the membrane [67]. Common extractants include di(2-ethylhexyl)phosphoric acid (D2EHPA), Aliquat 336, Cyanex extractants, and, more recently, ionic liquids (ILs) [72,73]. Plasticizers such as o-nitrophenyl octyl ether (ONPOE) and 2-nitrophenyl octyl ether (2-NPOE) are often added to improve membrane flexibility and facilitate the diffusion of REE-extractant complexes [74].

Similar to SLMs, PIMs combine the extraction and stripping steps of SX into a single membrane-based process. The adsorption or transport mechanism of REE ions through PIMs is also similar to that of SLMs [75]. At the feed-membrane interface, the extractant binds the target REE to form a complex. This complex diffuses through the polymer matrix toward the stripping phase. At the membrane-strip interface, the REE ion is released into the stripping solution through decomplexation. The regenerated extractant then diffuses back toward the feed solution, enabling continuous cyclic transport [76].

PIMs were developed to address the stability issue commonly observed for SLMs [45]. The immobilized polymer matrix in PIMs enhances mechanical integrity and improves long-term membrane stability by effectively retaining the extractant within the membrane [68]. As a result, common issues associated with SLMs, such as emulsion formation and solvent leakage, are addressed directly [77]. However, the immobilization of the extractant may result in reduced solute flux. Despite this trade-off, the mechanical properties of PIMs can be tailored to meet specific separation requirements and operating environments, including acidic or chemically harsh conditions [76,78]. REE selectivity can also be tuned by choosing appropriate polymer matrices, plasticizers, and extractants. For example, Oluwasola et al. [79] reported less than 7% solvent loss for a PVC-based PIM incorporating Bis(2-ethylhexyl) phosphate (HDEHP) after 48 h in acidic media (pH 2-4), demonstrating high membrane stability. In another study, Chen et al. [71] developed a PIM for separating Yb^3+^ and Lu^3+^ by incorporating the hydrophilic additive poly(vinyl alcohol-co-ethylene) (EVOH). Using Cyanex 727 as the extractant, they achieved a separation factor of 3.78, highlighting the role of membrane composition in tuning selectivity.

Several recent studies have demonstrated the effectiveness of PIMs for separating adjacent REEs (see Table 3). Huang et al. [75] investigated the selective transport of Lu^3+^ from solutions containing La^3+^ and Sm^3+^. They achieved a Lu^3+^ recovery of 91% after 36 h, while only 5% of Sm^3+^ was transported. In another study, Wang et al. [80] incorporated Cyphos IL 104 into a PIM to separate Lu^3+^ and Yb^3+^. The IL improved membrane hydrophilicity and permeability, but the separation factor reduced to 1.37. Nevertheless, these studies demonstrate that PIMs are a promising platform for the separation of adjacent REEs, although extractant selection remains crucial in achieving high selectivity.

Despite these advances, the separation of adjacent REEs remains challenging due to their highly similar ionic radii and electronic structures. For example, the ionic radii of La^3+^ and Lu^3+^ differ by only about 0.025 nm [81]. To further improve selectivity, ion-imprinted membranes (IIMs) have been proposed as an alternative to PIMs, offering enhanced discrimination between ions with very similar sizes.

**Table 3:** Representative studies on REE separation using PIMs.

Study	Membrane Type	Extractant	Plasticizer	Feed Solution	pH	Extraction (%)	Stripping/Recovery (%)
[75]	PVC	D2EHPA	-	La, Sm, Lu	1.5	-	Lu: 91
[71]	PVC + EVOH	Cyanex 727	-	Yb, Lu	3.5–4.5	SF_Lu/Yb_ = 3.78	-
[80]	PVC	Cyphos IL 104	-	Yb, Lu	5	SF_Lu/Yb_ = 1.37	-
[82]	CTA	D2EHPA	NPOE	La, Ce	-	-	La: 35, Ce: 65
[83]	CTA	Cyphos IL 104	NPOE	La, Y, Nd, Sm	4–5	-	Y: 100, Sm: 65, La: 55, Nd: 45

### 3.3. Ion-Imprinted Membranes

Ion-imprinted membranes (IIMs) are membranes engineered to selectively recognize and bind target ions through well-defined ion-recognition sites embedded within the membrane structure. These recognition sites are derived from crosslinked polymer networks synthesized in the presence of a template ion, known as ion-imprinted polymers (IIPs). Owing to the imprinting process, IIPs exhibit a strong affinity for the target ion while showing minimal interaction with competing ions, even when those ions have similar ionic radii and coordination characteristics [84,85]. To improve mechanical stability, reusability, and the availability of active binding sites, IIPs are typically immobilized on the membrane surface or incorporated within the porous polymer matrix [86].

**Figure 6:**
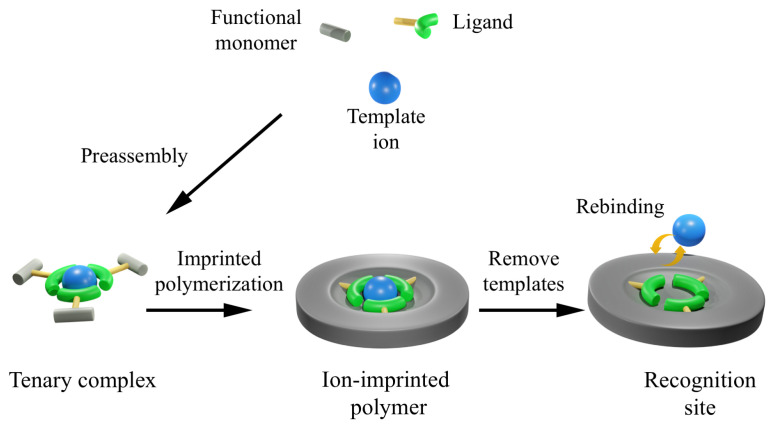
Illustration of the ion-imprinting process.

The ion-imprinting fabrication involves forming coordination complexes between a template ion and functional monomers, followed by polymerization and crosslinking. After the removal of the template ion, the remaining binding cavities are complementary in size, geometry, and coordination chemistry, enabling selective chelation [87] (see Figure 6). The template ion therefore plays a crucial role in defining the chemical and structural characteristics of these recognition sites [88]. During membrane fabrication, interactions between the template ion and functional monomers spatially organize the monomers, making the resulting membrane structure highly dependent on coordination chemistry [86].

Crosslinking plays another critical role in stabilizing the three-dimensional imprinted architecture by forming covalent bonds among functional monomers, thereby enhancing mechanical robustness and chemical resistance [89]. The ratio of monomer to crosslinker strongly influences the membrane’s morphology and porosity, directly influencing transport and binding performance [88]. Common crosslinkers include ethylene glycol dimethacrylate (EGDMA) [90], divinylbenzene (DVB) [91], and trimethylolpropane trimethacrylate (TRIM) [92].

Ion transport in ion-imprinted membranes (IIMs) is governed by recognition-based complexation at imprinted sites and is primarily controlled by the strength of ion-ligand interactions. For trivalent REEs, strong coordination with imprinted ligands typically leads to adsorption dominated transport. This behavior is characterized by slow adsorption/desorption kinetics, high selectivity, and low flux due to long residence times and significant desolvation penalties [93]. In principle, ions repeatedly bind and release from successive recognition sites across the membrane, which enables continuous permeation. However, this mechanism is rarely realized for REEs and has only been demonstrated in a limited number of highly optimized composite or hierarchically structured membranes [94]. As a result, most IIMs function primarily as selective adsorptive membranes rather than true carrier-mediated systems. In such systems, membrane regeneration and ion release can be achieved by adjusting pH, ligand concentration, or electrostatic conditions [93].

IIMs can be classified as filled, hybrid, free-standing, or composite, based on the incorporation mode of IIPs [84]. Filled IIMs are fabricated by physically incorporating IIP particles that have been ground into powder, enabling simple membrane preparation and potential multi-ion separation. However, the grinding process can damage imprinted sites and compromise separation performance [86]. Hybrid IIMs are produced by dispersing pre-synthesized IIPs within a polymer casting solution, followed by membrane formation via phase inversion. This approach improves stability and filler distribution, but may restrict access to some recognition sites [95]. Free-standing IIMs consist entirely of self-supporting imprinted polymer networks. This design avoids issues such as filler damage and buried recognition sites. However, the high crosslinking density required to maintain structural integrity often results in low porosity and limited mechanical strength [84,96]. Finally, composite IIMs feature a thin, surface-bound imprinted layer on a porous substrate created via in-situ polymerization, grafting, or interfacial polycondensation [97]. This architecture maximizes site accessibility, enhances selectivity and reusability, and enables tailoring of surface properties such as hydrophilicity and antifouling behavior [84,98].

Table 4 has summarized representative studies that demonstrate the potential of IIMs for separating adjacent REEs. For example, Chen et al. [99] fabricated yttrium ion-imprinted membranes using Cyanex 272 as both extractant and imprinting ligand, achieving strong selectivity for Y^3+^ in an aqueous REE mixture. The ion-imprinted membrane showed an adsorption capacity of 9.19 mg g^−1^ for Y^3+^, nearly three times that of the non-imprinted membrane (3.57 mg g^−1^). In comparison, the adsorption of competing Ho^3+^ and Er^3+^ was much lower, at 3.32 and 4.24 mg g^−1^, respectively. These results indicate that the imprinting cavities provide a selectively rebinding affinity for template Y^3+^ ions over the other interference ions. Similarly, Pan et al. [100] developed yttrium ion-imprinted electrospun membranes that exhibited high separation factors of 2.01 for Y/Ho and 1.77 for Y/Er, representing a substantial improvement over the corresponding non-imprinted membranes (1.24 and 0.85, respectively) and retained more than 86% of their initial flux after eight permeation cycles. Bio-based IIMs have also demonstrated promising performance. Rybak et al. [101] reported chitosan-modified IIMs capable of selectively adsorbing Nd^3+^ and Y^3+^ from coal fly ash extracts while retaining approximately 85% of their initial capacity after multiple regeneration cycles.

By integrating ion-imprinting chemistry with membrane technology, IIMs combine selective ion recognition and separation within a single platform, simplifying process design and enhancing overall efficiency [86,102]. Despite these advantages, several challenges still limit their industrial application for REE separation. Strong ion binding often results in low permeation flux and reduced process efficiency, while fouling by coexisting impurities decreases site availability and shortens operational lifetime. Additionally, complex and costly synthesis procedures, together with difficulties in achieving true transport rather than adsorption-dominated behavior, hinder large-scale implementation. To address these limitations, recent research progress includes the development of nanocomposite and hybrid membranes to improve antifouling properties [103], advanced ligand and template engineering to enhance selectivity among adjacent REEs [104], and hierarchical membrane architectures designed to increase flux while maintaining high selectivity [94].

**Table 4:** Summary of representative studies on REE separation using IIMs.

Study	Membrane Type	Imprinting Ligand/Functional Monomer	Target REE(s)	Feed Solution	pH	Performance
[99]	Y-IIM	Cyanex 272	Y	Aqueous REE mixture	≈4.5	Adsorption capacity 9.19 mg g^−1^ versus 3.57 mg g^−1^ for non-IIM.
[100]	Electrospun Y-IIM	Cyanex 272	Y	Aqueous Y/Ho/Er mixture	4–5	SF_Y/Ho_ = 2.01, SF_Y/Er_ = 1.77; only 13.8% flux loss after 8 cycles.
[101]	Chitosan-based IIM	Carboxyl and amino groups	Nd, Y	Coal fly ash leachate	5–6	Adsorption capacity of 85% after 5 cycles
[105]	Chitosan-PEG-PVA hybrid IIM	Carboxyl and amino groups	Nd	Aqueous REE mixture	5	SF_Nd/Eu_ = 3.47, SF_Nd/Dy_ = 3.72

### 3.4. Nanofiltration Membranes

**Figure 7:**
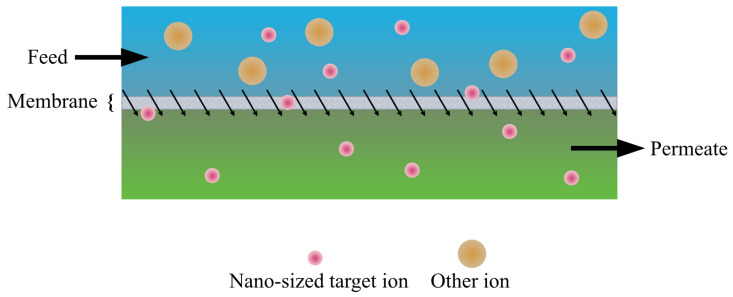
Schematic representation of a typical nanofiltration membrane.

Nanofiltration membranes (NFMs) are pressure-driven, semi-permeable membranes made from polymer thin films, most commonly crosslinked polyamide, supported on materials such as polysulfone or cellulose acetate [106]. Figure 7 illustrates a typical NFM structure. They are designed to separate solutes with molecular weights between 100 and 2000 Da without the need for phase change. A key characteristic of NFMs is their high rejection of multivalent ions, typically exceeding 99%, while allowing partial passage of monovalent ions (<70%). Additionally, NFMs can reject more than 90% of organic compounds whose molecular sizes exceed the membrane pore size [107,108,109,110]. These characteristics make NFMs as an important technology for liquid-phase separations [111,112,113].

In terms of the separation performance, NFMs lies between ultrafiltration membranes (UFMs) and reverse osmosis membranes (ROMs). Although ROMs provide high-purity separations, they require high operating pressures, making them more energy-intensive. UFMs, in contrast, operate at lower pressures but have larger pore sizes and lower rejection of undesired solutes. NFMs bridge this gap by offering moderate solute rejection with relatively high permeability and lower operating pressures [107]. However, operation in this transitional regime involves complex transport behavior governed by steric, electrostatic, and dielectric interactions, which remain an active area of research.

Despite their advantages, NFMs face several limitations, including membrane fouling, limited flux, and an inherent trade-off between selectivity and permeability [114,115]. To address these challenges, recent research has focused on incorporating nanomaterials into membrane matrices to enhance hydrophilicity, antifouling resistance, selectivity, and flux. Such modifications can also reduce energy consumption, chemical cleaning requirements, and overall operational costs [115].

NFMs are fabricated from polymeric materials using phase inversion or interfacial polymerization techniques [107]. Phase-inversion NFMs are structurally asymmetric, meaning they exhibit a nonuniform pore distribution throughout the membrane. They are produced from single polymer component such as cellulose acetate or poly(ether)sulfone. In contrast, interfacial polymerization produces thin-film composite membranes, comprising a dense selective layer formed on a porous ultrafiltration support. Common polymers include aromatic polyamides, sulfonated polysulfones, and poly(piperazine amide). More recently, highly cross-linked polymer networks have been developed to improve membrane stability under extreme pH conditions, elevated temperatures, and exposure to organic solvents [110,113]. NFMs also contain ionizable functional groups, resulting in surface charges that depend on solution pH, with most NFMs exhibiting a net negative charge at neutral pH. For example, Zhao et al. [109] showed that ion-functionalized nanofiltration membranes exhibit increasingly negative surface charge with increasing pH due to deprotonation of acidic functional groups, which strongly influences the rejection and separation behavior of multivalent metal ions.

Separation in NFMs is governed by a combination of size-based and electrostatic interactions between solutes and the charged membrane surface, with mass transport occurring via both convection and diffusion [43]. These effects manifest through three primary mechanisms: steric exclusion, Donnan exclusion, and dielectric exclusion. Steric exclusion arises from size-based sieving, whereby solutes larger than the membrane pores are rejected while smaller species permeate [116]. Transport associated with steric exclusion is driven by pressure gradients that induce convective flow, as well as concentration gradients that promote diffusive transport [111]. Donnan exclusion results from electrostatic interactions between charged solutes and the charged membrane surface, leading to the formation of a Donnan potential at the membrane-solution interface, which repels co-ions and attracts counter-ions while maintaining electroneutrality [117,118]. Dielectric exclusion arises when ions encounter an energy barrier associated with changes in solvation energy upon entering the lower-dielectric membrane pores [110]. Together, these mechanisms govern the selectivity of NFMs toward ions of different charge, size, and hydration energy.

Table 5 have summarized studies that demonstrated the application of NFMs in REE separation. López et al. [108] investigated REE transport through two NFMs with different active layers, a double layer poly(piperazinamide)/polyamide membrane (Desal DL) and a sulfonated polyethersulfone membrane (HydraCoRe 70pHT), for recovery from acid mine waters. They reported that separation performance was strongly influenced by REE speciation and by the chemical properties of the membrane active layer. Similarly, Murthy and Choudhary [119] evaluated Nd^3+^ separation from aqueous solutions and observed that rejection increased with applied pressure and feed flow rate but decreased with increasing feed concentration. Rejection was also strongly pH-dependent, confirming the role of membrane charge in ion transport. In a related study, Murthy and Gaikwad [120] examined the separation of Pr^3+^ using an NF-300 membrane under varying operating conditions. Separation efficiency increased with applied pressure (2–10 bar) and cross-flow rate (4–16 L min^−1^) but decreased with increasing feed concentration. Within the investigated concentration range, maximum separations of 89.07% and 84.20% were achieved, which increased to 99.28% and 99.30% upon the addition of ethylenediaminetetraacetic acid (EDTA) and diethylenetriaminepentaacetic acid (DTPA), respectively. These results underscore the significance of solution chemistry and complexation in improving REE rejection by NFMs.

Recent studies have confirmed the effectiveness of NFMs for REE recovery from leachates [122] and for separating REEs from competing non-REE ions, including actinides and alkali metals [121,123]. However, studies specifically targeting the separation of adjacent REEs using NFMs remain scarce. This gap underscores the need for further investigation into the selective separation of chemically similar REEs using nanofiltration-based techniques.

### 3.5. Electrodialysis Membranes (EDMs)

**Figure 8:**
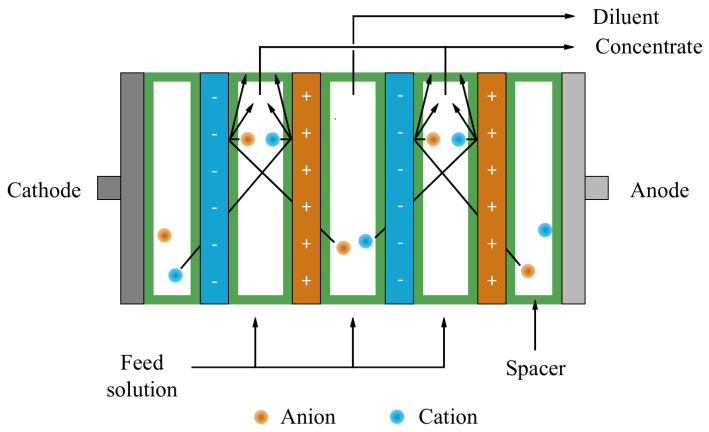
Schematic representation of a typical electrodialysis membrane.

Electrodialysis membranes (EDMs) are specialized ion-exchange membranes (IEMs) that use an electrical potential difference to extract and separate target metal ions. IEMs consist of polymer networks with fixed-charge functional groups that provide ion selectivity through membrane permselectivity [124]. An electrodialysis membrane (EDM) system consists of alternating cation-exchange membranes (CEMs) and anion-exchange membranes (AEMs) arranged between dilute and concentrate compartments, with electrode chambers positioned at each end [125]. As shown in Figure 8, in an electrodialysis separation process, when a direct current is applied, cations migrate toward the cathode through CEMs, while anions migrate toward the anode through AEMs [126]. This process generates both ion-depleted and ion-enriched streams [127]. This electrically driven separation mechanism makes EDMs attractive for REE separations. This is because variations in REE complexation can alter ionic charge and mobility, enabling selective transport even when the ions have similar sizes and hydration energies [128].

EDMs are made in polymer matrices to ensure mechanical stability and low electrical resistance. Common materials include polystyrene-divinylbenzene copolymers containing ionogenic functional groups dispersed within an inert binder [129,130]. Nonwoven backing layers are added to improve membrane durability and lifespan [131]. Spacers are inserted between membranes to form flow channels and maintain uniform hydrodynamics in the electrodialysis stack [126]. Generally, EDMs are stacked in 5-10 cell pairs to enhance concentration gradients and separation efficiency [132]. Additional design modifications, such as surface treatments and rinse compartments, are employed to mitigate fouling, suppress precipitation, and isolate electrode reactions that could compromise membrane performance [133].

As summarized in Table 6, several studies have shown that EDMs can effectively separate REEs, especially when combined with chelating agents. For instance, Takahashi et al. [134] explored the separation of La^3+^ and Nd^3+^ from multicomponent REE solutions using lactic acid and EDTA. They reported separation factors as high as 100 for La/Y systems, attributed to differences in REE-ligand stability constants that affect free-ion concentration under the electric field. Mosadeghsedghi et al. [135] further demonstrated the separation of Ce^3+^ and Yb^3+^, showing that the addition of EDTA led to improved separation factors. They also confirmed that higher voltage improved efficiency, highlighting the key role of the electric field.

More recently, Mosadeghsedghi et al. [136] reported the separation of light and medium REEs from binary and tertiary mixtures using various chelating agents like EDTA, DCTA, HEDTA, and DTPA. They achieved separation factors up to 42 (≈20 times higher than what traditional SX typically provides) [137,138]. In another study, Li et al. [125] achieved a Sc^3+^ recovery of 99.52% from dilute solutions with competing ions, demonstrating the energy efficiency of EDM processes with low energy consumption (0.26 kWh m^−3^).

Despite their potential, EDMs still face challenges such as membrane fouling, degradation of ion-exchange groups, and polymer aging due to electric current, Joule heating, hydrodynamic shear, and chemical exposure. These issues can lower membrane performance over time and require frequent replacement. Recent studies have suggested solutions like feed pretreatment, current mode optimization, hydrodynamic condition control, membrane surface modification, crosslinking, and regeneration techniques to mitigate these limitations [139].

In summary, several studies indicate that EDMs are a promising alternative for REE separation, especially when combined with chelating agents. However, most work has focused on separating distant REEs or recovering individual REEs, leaving a gap in research on the separation of adjacent REEs, where chemical similarities are more significant. This highlights the need for focused research on using EDMs in this area.

### 3.6. Metal-Organic Framework (MOF) Membranes

Metal-organic frameworks (MOFs) are crystalline, porous materials that form through the self-assembly of metal ions or clusters coordinated with organic ligands [140]. They feature diverse, tunable structures, periodic pore networks, and high porosity, with free volumes reaching up to 90% in some ultraporous structures. MOFs have internal surface areas exceeding 6000 $m^{2}g^{-1}$) [47,141,142]. Their regular pore networks and the ability to modify internal surfaces and metal sites allow for the creation of specific binding environments for target guest ions or molecules. This facilitates precise control over size, shape, and coordination selectivity [141,143]. These attributes make MOF-based membranes effective materials for selective ion and molecular transport.

Ion transport in MOF membranes involves multiple factors: steric (size) sieving, electrostatic interactions, coordination/chelation within pores, dehydration/desolvation, and energy barrier modulation [144,145]. REE ions migrate by hopping between coordination sites, diffusing along water-mediated paths, and traveling through connected pores [146,147,148]. Size-based sieving alone is insufficient for adjacent REEs, which differ in ionic radii by only about 0.02 nm. Selectivity, therefore, mainly depends on differences in the coordination chemistry, dehydration energy, binding affinity, and local electrostatic environments [149,150]. Molecular simulations show that variations in ion-membrane binding energies significantly affect REE selectivity, even in MOFs with large pores [151]. Dehydration energy barriers are significant for trivalent REEs, as the energy cost of removing hydration shells can limit transport. Ions with less stable hydration shells can enter MOF pores more easily, favoring their transport [149]. Additionally, functional groups in MOF channels can coordinate with REE ions, influencing their adsorption and residence times [47]. Further enhancement of selectivity can be achieved via electrostatic effects arising from charge distribution within pores, especially in mixed feeds with competing ions differing only in charge density and hydration entropy [152].

**Figure 9:**
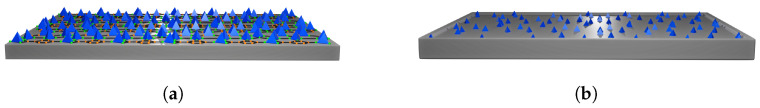
Illustration of a typical MOF-based membrane: (**a**) Pure MOF (**b**) MMM. Schematic key: Blue triangles represent MOF crystals/particles (porous fillers providing selective transport pathways). Orange lines/grid in (**a**) depict the interconnected MOF framework/lattice itself. In (**b**), the MMM shows MOF particles (blue) dispersed within a continuous polymer matrix (gray/uncolored background), with no orange coloring used to emphasize the distinct polymer phase as the continuous binder (unlike the MOF-only network in (**a**)).

There are two main types of MOF membranes: pure MOF membranes and MOF-polymer hybrid membranes, also known as mixed-matrix membranes (MMMs) (Figure 9). Pure MOF membranes feature a continuous crystalline layer grown or deposited on a porous support [153,154]. In these membranes, the MOF’s pore network governs transport, allowing orderly diffusion pathways and precise molecular sieving. Pure MOFs, like Zr MOFs, exhibit excellent thermal and chemical stability, and are resistant to fouling and swelling [155,156,157]. However, challenges such as film brittleness, defect control, and large-area fabrication limit their practical use [154]. In contrast, MMMs comprise dispersed MOF particles in a continuous polymer matrix, combining MOFs’ selectivity with the strength of polymers [158]. This design addresses some limitations of pure MOF membranes, particularly brittleness and scalability, while maintaining good separation performance. Nonetheless, MMMs face issues such as filler agglomeration and poor filler-polymer compatibility, which may create nonselective voids [159]. Addressing these interfacial issues is an ongoing research focus [159].

As summarized in Table 7, recent research highlights the potential of MOF-based membranes for REE separation. Lee et al. [160] studied the adsorption of La^3+^, Ce^3+^, Nd^3+^, Sm^3+^, and Gd^3+^ on functionalized MIL-101 frameworks, showing that separation relies on surface complexation and electrostatic interactions. Song et al. [47] reported high separation factors (up to 908 for La^3+^/Nd^3+^ and 543 for Ce^3+^/La^3+^) using an ion-microporous MOF (ATZ-BTC-Zn) for adjacent REE separation in tailings wastewater. Similarly, Wu et al. [161] achieved selectivities up to 10,000 for Ce^3+^/Lu^3+^ with a Zn-BTC MOF/nanoporous graphene hybrid membrane. More recently, Hu et al. [162] designed an MOF (NCU-1) that combined dense uncoordinated carboxyl groups and triazole N atoms to create a highly responsive REE nanotrap. Using binary models, they achieved separation factors of 796 for Pr/Lu and 273 for Nd/Er in a single step. These studies demonstrate that MOF nanochannels and functionalized frameworks can distinguish chemically similar REEs by exploiting differences in size, hydration energy, coordination affinity, and engineered pore environments. As a result, separation factors far exceeding those of conventional polymeric membranes can be achieved, although such extreme values are typically obtained under idealized laboratory conditions.

Despite their tunable properties, MOF membranes still have limitations. Many frameworks lack hydrolytic stability in acidic or aqueous environments [163]. Some flexible or interpenetrated MOFs may lose porosity or collapse upon activation [164]. These challenges highlight the need for careful framework selection, chemical stabilization, and membrane engineering to ensure long-term performance in practical applications.

Although MOF-based membranes stand out for their tunable pores and functionality, traditional inorganic membranes, such as ceramic (alumina, titania, zirconia), silica-based, zeolite-based, and inorganic ion-exchange types, have also been explored for REE separations. These conventional materials primarily use size- or charge-based rejection, surface charge selectivity, and (in zeolites) adsorption/kinetic/diffusion effects. They offer excellent chemical, thermal, and mechanical stability, making them suitable for harsh environments like acidic leachates [165]. Despite these advantages, their ability to selectively separate REEs remains limited without functionalization or the use of complexing agents. For instance, in tests with synthetic acid mine waters (pH 1.0-1.5 containing Al^3+^, Fe^3+^, and REE^3+^ such as La, Pr, Nd, Sm, Dy, Yb), a TiO_2_ ceramic NF membrane achieved metal rejections of only 5–30% (highest for trivalent species), while a polymeric MPF-34 membrane provided approximately 80% metal rejection (independent of major ion concentrations). Additionally, selectivity among REEs or against competing metals remained low due to speciation effects and lack of targeted functionalization [166]. MOFs thus represent a key, innovative subclass of inorganic membranes for precise REE targeting.

**Table 7:** Summary of representative studies on REE separation using MOF-based membranes.

Study	Membrane Type	MOF/Filler	Target REEs	Feed Solution	Performance
[47]	Pure MOF membrane	ATZ-BTC-Zn	La/Nd, Ce/La	Tailings wastewater	SF_La/Nd_ = 908, SF_Ce/La_ = 543
[160]	Pure MOF (adsorptive)	Functionalized Cr-MIL-101	La, Ce, Nd, Sm, Gd	Aqueous REE solutions	Selectivity of ≈90% for Gd among transition metals
[161]	MMM	Zn-BTC MOF/nanoporous graphene	Ce/Lu	Binary aqueous solution	Selectivity up to $10^{4}$
[162]	Pure MOF (nanotrap)	NCU-1 (two-fold interpenetrated MOF with carboxyl and triazole groups)	Pr/Lu, Nd/Er	Tailings wastewater	SF_Pr/Lu_ = 796, SF_Nd/Er_ = 273
[167]	MMM	Cr-MIL-PMIDA in sulfonated poly(ether ketone)	Eu	Aqueous solution	Operated for 12 days without degradation

### 3.7. Hybrid Membrane-Based Separations

The previous sections discussed various membrane technologies used for REE separation, summarized in Table 8. While each type of membrane has unique advantages, their individual use is often constrained by trade-offs in selectivity, flux, stability, and fouling resistance. To address these issues, recent research is shifting toward hybrid membrane systems that combine different materials and transport mechanisms within a single architecture, enhancing both separation performance and durability. For instance, Chen et al. [168] created zwitterionic IIP brushes on mixed-matrix membranes (MMMs), combining molecular recognition with improved hydrophilicity and antifouling properties. This hybrid design achieved separation coefficients of 2.16 for Y^3+^/Ho^3+^ and 2.77 for Y^3+^/Er^3+^, significantly higher than those for MMMs alone, showcasing the benefits of integrating ion imprinting with matrix reinforcement. Similarly, Lv et al. [169] developed a multilayer hybrid membrane using two-dimensional vertical heterojunctions of graphene oxide, ZIF-8, and polydopamine (GO/ZIF-8/PDA). This design enabled size sieving, coordination affinity, and interfacial charge effects to selectively transport smaller hydrated lanthanide ions while excluding larger hydrated Sc^3+^. The selectivity ratio of approximately 68.7 for Sc^3+^ over other REEs highlights the potential of well-designed hybrid interfaces to overcome the intrinsic limitations of single-component membranes.

In addition to hybridization at the material level, emerging evidence shows that membrane performance in complex REE separations is significantly influenced by system-level design. Factors such as chemical stability, operating mode, process configuration, and integration with auxiliary operations can be as crucial as the membrane’s intrinsic selectivity and permeability. For example, combining nanofiltration with diafiltration has been shown to enable separations that would be difficult or inefficient using single-pass nanofiltration alone, especially for systems involving multivalent and trace ions. This process-level hybridization can leverage phenomena such as negative-ion rejection and composition-dependent transport to improve purification efficiency as well as selectivity [170,171].

**Table 8:** Comparison of membrane-based techniques for rare earth element (REE) separation.

Technique	Optimum Separation Factor (β)	Optimum Recovery/ Extraction/Rejection (%)	Carrier/Operating Conditions	Target REEs
SLM [58,59,172]	–	58–98.5	D2EHPA, P507 (EHEHPA), TOPO/TBP	Y, Dy, Nd, La
PIM [71,75,82]	1.3–3.8	35–91	Cyphos IL 104 or Cyanex 727 in PVC/ PVDF/EVOH	Lu/Yb, La, Lu
IIM [99,100,101,105]	1.7–3.7	–	IIP matrices (chitosan, electrospun fibers); Cyanex 272 or carboxyl/amino ligands	Nd/Eu, Nd/Dy, Y/Ho, Y/Er
NFM [108,120]	–	92–99	Polyamide or PEI-modified membranes; chelant-assisted (EDTA/DTPA)	La, Nd, Pr
EDM [125,134,136]	2–42 (up to 100 for distant pairs)	up to 99	CEM/AEM stacks; EDTA/DTPA chelation	Ce/Yb, La/Y, Nd, Gd
MOF membrane [47,161]	2–10^3^ (in optimized systems)	–	Pure MOF films or MMMs; functionalized pores	Ce/La, La/Nd, Ce/Lu

Together, these findings underscore the need for a holistic approach to hybrid membrane-based separations, in which membrane materials, transport phenomena, and system design are considered concurrently rather than in isolation.

### 3.8. Summary of Membrane-Based Separation

Membrane-based technologies offer a range of strategies for REE separation, characterized by distinct transport and selectivity mechanisms. SLMs and PIMs utilize carrier-mediated transport for high extraction efficiencies through selective complexation. However, they often face issues with carrier loss, long-term stability, and limited separation factors for adjacent REEs. IIMs feature molecular-level recognition with templated binding sites, enhancing selectivity for specific REEs. Yet, their strong binding can restrict permeation flux. NFMs rely on exclusion and electrostatic effects for high throughput and operational simplicity. However, they struggle to distinguish between adjacent REEs that differ only slightly in ionic size and hydration. EDMs utilize an electric field to drive ion migration, achieving high separation factors when combined with complexing agents. This, however, can increase energy demands and lead to membrane degradation. Emerging MOF membranes offer a promising shift, featuring tunable pore structures and coordination environments that allow selectivity based on dehydration energetics and binding affinity rather than size alone. Nonetheless, they still face challenges with hydrolytic stability and scalable fabrication. Collectively, these membrane platforms highlight the fundamental trade-offs between selectivity, flux, stability, and process complexity.

These trade-offs become particularly pronounced when membrane systems are applied to chemically complex and compositionally realistic feed streams. In the REE separation, feed streams contain large amount of competing ions, resulting in interactions, such as competitive binding, ion-ion association, charge screening, and solution-phase complexation, that profoundly influence ion fluxes and separation factors. These effects will result in opposite separation trends observed in single-ion experiments. Therefore, there is a crucial need for systematic studies on multicomponent transport behavior under realistic conditions. This includes combining experimental and modeling approaches to clarify the dominant interaction mechanisms governing selectivity.

## 4. Techno-Economic Considerations for Membrane-Based REE Separations

While membrane-based processes show strong potential for REE separation at the laboratory and pilot scales, their successful transition to commercial applications depends critically on techno-economic performance and scalability. Therefore, emerging membrane technologies should be assessed against established technologies such as solvent extraction (SX), which remains the dominant industrial benchmark for REE separation due to its proven scalability, high throughput, and established cost structure.

Despite its industrial maturity, SX processes incur substantial operating costs related to extractant consumption, chemical reagents, and waste management. A representative techno-economic assessment (TEA) by Argumedo et al. [173] indicated that organic extractants cost approximately $8.3 ${\mathrm{kg}}^{-1}$ of ash processed, with significant reagent usage including nitric acid ($0.60 ${\mathrm{kg}}^{-1}$), sodium hydroxide ($0.32 ${\mathrm{kg}}^{-1}$), hydrochloric acid ($0.115 ${\mathrm{kg}}^{-1}$), and sodium metabisulfite ($0.28 ${\mathrm{kg}}^{-1}$). Additionally, waste handling increased costs, with hazardous wastewater disposal at around $0.019 ${\mathrm{kg}}^{-1}$ and leached solid waste disposal at approximately $0.010 ${\mathrm{kg}}^{-1}$. Thus, the capital expenditure (CAPEX) and operational expenditure (OPEX) per kg of ash were estimated to be $0.29 and $0.145, respectively, with recovered rare earth oxide having a value of about $0.129 ${\mathrm{kg}}^{-1}$. These figures highlight the chemical and waste-intensive nature of SX-based REE separation, providing a quantitative baseline for evaluating membrane-based technologies, which typically require fewer reagents and produce less hazardous waste.

Recent dedicated TEAs of membrane-based REE separations remain scarce. Most TEAs reported in the literature for REE separation and recovery focus on conventional approaches such as solvent extraction, leaching, and precipitation [174,175,176,177], with only a few preliminary studies exploring membrane-based approaches. For example, a 2018 TEA evaluated a hybrid microfiltration-nanofiltration process for REE recovery from coal fly ash, identifying membrane lifetime, fouling control, and energy demand as key economic drivers. However, the separation of individual REEs was not addressed and detailed, system-level cost assessment was not provided [178]. Membrane and module costs also account for a substantial portion of CAPEX in these systems. At an industrial scale, polymeric membranes generally cost $50–200 $m^{-2}$, while advanced membranes like mixed-matrix can range from $5–50 $m^{-2}$, or in some cases up to $500 $m^{-2}$. Ceramics can reach up to $3000 $m^{-2}$, depending on their complexity and module configuration [179,180]. In addition to upfront costs, the expected lifetime of membranes (typically between 2 and 8 years in TEA models) significantly affects replacement frequency and overall ownership costs [181,182].

For emerging inorganic membranes like MOFs, techno-economic uncertainty is high. While MOF membranes offer excellent tunability and selectivity, their high synthesis costs, challenges in scaling up, and uncertain long-term stability limit their competitiveness compared to standard polymeric nanofiltration or ion-exchange membranes when measured against SX. Thus, MOF-based membranes are better suited as selective layers in hybrid or multistage separation systems rather than as direct replacements for solvent extraction.

It is essential to note that comparing membrane-based processes to SX is context-dependent. This is because operating costs are heavily influenced by factors such as feed composition, REE concentration, operating conditions, process boundaries, and scale. Additionally, the economic value of the recovered product depends on the specific REEs separated as well as prevailing market prices. Furthermore, although absolute cost estimates may evolve over time due to fluctuations in chemical prices, energy costs, and market conditions, the relative cost drivers and comparative trends in these studies remain instructive for assessing process feasibility.

In summary, existing techno-economic evidence suggests that while membrane-based REE separations are economically viable, they are unlikely to replace solvent extraction on a one-to-one basis in the near term. Instead, their best economic potential lies in hybrid configurations, selective pre-concentration, and the treatment of complex or dilute feed streams, where reduced chemical consumption and lower waste generation can offset higher membrane and module costs. Thus, continued integration of detailed TEA alongside experimental membrane development will be essential to guide future progress toward economically viable REE separation technologies.

## 5. Outlook for Advanced Hybrid Membrane Systems

Building on recent progress in hybrid membrane materials, transport modeling, and process integration, future research should focus on designing hybrid and hierarchically engineered membrane systems that integrate multiple separation mechanisms, along with staged separation architectures. One effective strategy is to divide functions across different stages. The order of these stages should be based on feed characteristics, such as concentration, volume, and impurity profile. A common and beneficial approach is to begin with preconcentration and impurity removal using nanofiltration (NFM) or electrodialysis (EDM). These processes can significantly increase REE concentrations, e.g., tripling levels from dilute sources such as coal fly ash or e-waste leachates. They can also reject competing monovalent ions, reduce feed volume, and partially remove multivalent impurities. This will simplify the feed composition and minimize competitive effects or fouling in downstream high-selectivity membranes [178].

After preconcentration, the stream can be processed by a high-selectivity hybrid membrane, such as UiO-66-NH_2_-based ion-imprinted nanocages integrated onto phosphonyl-functionalized SiO_2_-PVDF nanofiber membranes. This configuration enables partial resolution of adjacent REEs. For example, Xu et al. [183] used such MOF-templated nanocages with amino and phosphate groups for targeted Nd^3+^ recognition. They achieved a maximum adsorption capacity of 66.5 mg g^−1^, separation factors of 2.43 (Nd/Pr), 2.95 (Nd/Sm), and 3.24 (Nd/Dy), as well as permeation selectivities up to 7.94 (Nd/Dy). The system showed excellent regenerability (94.8% capacity retention after 10 cycles) and antifouling properties suitable for complex flowing phases. Such staged configurations (preconcentration first) reduce downstream competition, allowing advanced hybrids to approach their intrinsic selectivity limits more effectively.

Membrane technologies can further enhance traditional solvent extraction (SX) to improve efficiency and reduce waste generation. Membrane solvent extraction (MSX) uses supported membranes to immobilize organic extractants (e.g., D2EHPA or TODGA) for non-dispersive contact. This minimizes emulsion formation, solvent loss, and chemical use compared to conventional SX, allowing for continuous operation and high recovery rates (>95%) with exceptional purity (>99.5 wt% REE oxides) from various e-waste feeds [184,185]. Integrating upstream preconcentration through nanofiltration can also enhance subsequent MSX recoveries by 10% for total REEs and 30% for heavy REEs in single-stage operations. This setup also facilitates acid recovery from permeate streams, making the process more environmentally friendly and cost-effective [186]. These hybrid integrations provide practical routes to minimize environmental impact, chemical usage, and operational complexity while leveraging the strengths of both membrane and traditional methods.

## Figures and Tables

**Table 2:** Summary of representative studies on REE separation using SLMs.

Study	Membrane Type	Carrier	Feed Solution	pH	Extraction (%)	Stripping (%)
[59]	HFSLM	D2EHPA	WPM leach	2	Nd: 58.6, Dy: 98.5, Pr: 85.6	Nd: 63.1, Dy: 15.2, Pr: 56.3
[62]	FSHSLM	D2EHPA	AMD	2–4	60-82 (total REEs)	-
[60]	HFSLM	2-EHPA	Aqueous REE mixture	4.5	Nd: 98	Nd: 95
[61]	HFSLM	DNPPA	Monazite leach	4.5	Nd: 98	Nd: 95
[58]	HFSLM	Cyanex 272 + TBP	Aqueous REE mixture	5	Y: 70	Y: 55

**Table 5:** Representative studies on REE separation using NFMs.

Study	Membrane Type	Active Layer/Material	Target REE(s)	Feed Solution	pH	Performance
[119]	NF-300	Polyamide TFC	Nd	Synthetic aqueous solution	6–10	85–90% rejection; 99.45% with EDTA.
[120]	NF-300	Polyamide TFC	Pr	PrCl_3_ aqueous solution	6	89.07% rejection; 99.28% (EDTA), 99.30% (DTPA).
[121]	Positively charged NFM	Modified polyamide	La	REE-alkali metal mixture	2.7	90–99% rejection.
[122]	OGCN-modified NFM	Polyamide + O-doped g-C_3_N_4_	Mixed REEs (La, Y, Nd)	Leachate solution	3–5	>96% REE rejection.

**Table 6:** Summary of representative studies on REE separation using EDMs.

Study	Chelating Agent	Target REE(s)	Feed Solution	Operating Mode	Performance
[125]	None reported	Sc	Dilute aqueous solution	Constant voltage	Recovery: 99.52%; Energy: 0.26 kWh m^−3^
[132]	Various ligands	REE mixtures	Industrial-relevant streams	Stacked cell pairs	Enhanced concentration gradients
[134]	EDTA, lactic acid	La/Y; Nd/Gd/Y	Synthetic aqueous mixtures	Constant current	SF_La/Y_ up to 100
[135]	EDTA	Ce/Yb	Binary aqueous solution	Constant voltage	SF_Ce/Yb_: 1.44 (no EDTA), 2.60 (with EDTA)
[136]	EDTA, DCTA, HEDTA, DTPA	Light and medium REEs	Binary and tertiary mixtures	Constant voltage	SF up to 42

## Data Availability

No new data were created or analyzed in this study. Data sharing is not applicable to this article.

## Abbreviations

The following abbreviations are used in this manuscript:

AMD	Acid mine drainage
ATZ	3-Amino-1,2,4-triazole
BTC	1,3,5-Benzenetricarboxylate
CTA	Cellulose triacetate
D2EHPA	Di(2-ethylhexyl)phosphoric acid
DNPPA	Di-nonyl phenyl phosphoric acid
EDM	Electrodialysis membrane
EDTA	Ethylenediaminetetraacetic acid
EHEHPA	2-Ethylhexyl phosphonic acid mono-2-ethylhexyl
2-EHPA	Di(2-ethylhexyl)phosphoric acid
EVOH	poly(vinyl alcohol-co-ethylene)
FSHSLM	Flat-sheet supported liquid membrane
HEDTA	N-(2-Hydroxyethyl)ethylenediaminetriacetic acid
HFSLM	Hollow-fiber supported liquid membrane
IIM	Ion-imprinted membrane
IL	Ionic liquid
MMM	Mixed-matrix membrane
MOF	Metal-organic framework
MRI	Magnetic resonance imaging
MST	Membrane separation technologies
NFM	Nanofiltration membrane
2-NPOE	2-Nitrophenyl octyl ether
ONPOE	o-Nitrophenyl octyl ether
PDA	Polydopamine
PEI	Polyethyleneimine
PIM	Polymer inclusion membrane
PVDF	Poly(vinylidene fluoride)
PVC	Poly vinyl chloride
REE	Rare earth element
SF	Separation factor
SLM	Supported liquid membrane
SX	Solvent extraction
TBP	Tributyl phosphate
TODGA	Tetraoctyl diglycolamide
TOPO	Trioctylphosphine oxide
WPM	Waste permanent magnet
ZIF	Zeolitic imidazolate framework
β	Separation coefficient
